# Debridement, antibiotics and implant retention (DAIR) following hip and knee arthroplasty: results and findings of a multidisciplinary approach from a non-specialist prosthetic infection centre

**DOI:** 10.1308/rcsann.2023.0076

**Published:** 2023-11-20

**Authors:** F Awad, J Boktor, V Joseph, MH Lewis, C Silva, S Sarasin, PM Lewis

**Affiliations:** ^1^ Prince Charles Hospital, UK; ^2^ University of South Wales, UK

**Keywords:** DAIR, PJI

## Abstract

**Introduction:**

Prosthetic joint infection (PJI) is a catastrophic complication following arthroplasty surgery. Recently a debridement, antibiotics and implant retention (DAIR) procedure has gained popularity for PJI where a thorough debridement, irrigation and modular component exchange is undertaken.

**Method:**

We present the outcome for DAIR, data collected prospectively, in a busy orthopaedic unit but not one specialising in PJI. All patients with PJI were included without loss of data or patients from 2012 to 2018 with a minimum follow-up of 5 years.

**Results:**

Four total knee replacements, 17 total hip replacements, one revision total hip replacement and three hip hemiarthroplasties are included with an average duration from onset of symptoms to the DAIR procedure of 11 days (range 1–22 days). *Staphylococcus aureus* (24%) and *Staphylococcus epidermidis* (32%) were the most common causative organisms, and the most common antibiotic regimens included intravenous teicoplanin and flucloxacillin. Average follow-up was 67 months (range 9–104 months). Only four patients went on to require revision surgery. An analysis of midterm patient outcome measures for 6 of the total hip replacement (THR) DAIR patients were compared with a database of 792 THRs (with a minimum two-year follow-up) carried out by the same surgeon revealed no significant difference in Oxford hip scores at one-year post-surgery (OHS DAIR 36.2 vs 39 for control group).

**Conclusion:**

This study includes 25 consecutive patients treated with DAIR with only one reinfection, with a mean follow-up period of 5 years. Using a strict protocol, DAIR appears to offer a successful treatment strategy for the management of early PJI.

## Introduction

Basic principles in surgery usually develop from experience over many years of practice. One such principle includes the fact that any foreign body in the human body, once infected, will require its complete and radical removal for infection eradication and healing to occur.^1^ Only recently this fact has had to be re-learnt with experience of mesh used for urinary incontinence and hernia repairs, where the use of mesh gained popularity only to find that once infected, every piece of mesh has to be removed, with significant and serious consequences.^1^ Similarly, experience in vascular surgery has established that an infected Dacron or PTFE graft, once infected, needs its complete removal. Even when the graft is removed in total and replaced by a new and/or rifampicin antibiotic-soaked graft, reinfection and potential death can occur due to catastrophic bleeding from anastomotic sites.^1^ In general, therefore, it would appear that all foreign material once infected has to be removed for long-term infection eradication.

In orthopaedic surgery, total hip replacement (THR) and total knee replacement (TKR) surgeries account for over 160,000 operations per year with THR described as the ‘operation of the century’ due to quality-of-life improvements.^1^ However prosthetic joint infection (PJI), although rare (approximately 1%),^2^ is catastrophic and potentially life threatening. In England and Wales in 2018, 17% of THR revisions and 25% of TKR revisions were performed for infection.^2^ For many years, established treatment for PJIs consisted of removal of all foreign material followed by a thorough debridement of the joint space, sometimes the insertion of a spacer and the patient left often for months until a second stage revision is undertaken. Inevitably, such surgery requires multiple and prolonged hospital stays, and not infrequently transfer to specialist centres, and the procedure is associated with significant morbidity/mortality.^2^

Recently an alternative and seemingly counterintuitive procedure has been developed. Described as a debridement, antibiotics and implant retention (DAIR) procedure, this entails debridement of the articulation and antibiotic treatment but with **some implant retention**, seemingly a procedure doomed to fail as it has in the other specialties described above. Specifically, the DAIR procedure involves a thorough debridement of the joint cavity, exchange of modular components and if successful, no requirement for a two-stage procedure. The advantage if successful is a reduction in patient morbidity including multiple surgeries, mortality, treatment costs and transfer to specialist centres.

So has the DAIR procedure to date proved to be the second best ‘operation/treatment of the century’? A review of the most recent orthopaedic literature concerned with DAIR has shown that findings and conclusions are varied and often contradictory. For example, in one paper, following a literature review of 33 studies involving 1,266 patients, the authors concluded ‘DAIR efficacy and indications are controversial’.^3^ In another, DAIR is ‘widely accepted’,^4^ while another study concludes ‘the optimum timing for DAIR is unclear’.^4^ Another states ‘joint infection is an annoying problem’,^5^ whereas another states that PJI is a ‘devastating complication of hip arthroplasty’.^6^ Another concludes ‘DAIR procedures offer similar or better results than a 2 stage procedure’.^7^ These studies have been collected from a wide variety of units, some specialised and some using national registries,^8^ making comparisons difficult. One paper even concluded that a *Staphylococcus aureus* infection (notorious for its persistence^9^) has better results for DAIR than for less virulent bacteria. Lastly, the timing for DAIR intervention has varied from days to months following the primary joint insertion, seemingly ignoring the fact that once a biofilm has developed, failure is inevitable and a two-stage procedure is essential for eradication of the PJI.^10^

This study reports data collected prospectively from our unit, which has a speciality interest in THR and TKR and a large turnover of patients as reflected in recent publications.^7^ Our management of PJI is restricted to a very strict protocol agreed prior to study commencement. It involves only two surgeons who have either personally undertaken the index joint replacement or have accepted a referral from colleagues from other hospitals. The DAIR is then undertaken by either consultant for all operations. Furthermore, our protocol for PJI (hip and knee) requires that the DAIR is undertaken within three weeks of the joint replacement or acute onset of symptoms. Bacterial sample data and number of samples collected are all reviewed by one microbiologist (with a major interest in PJI) along with a tailored antibiotic regime and the treatment length of time. All patient data have been collected in an identical fashion, both pre- and post-DAIR, and results are presented in detail below. No patients were lost to follow-up.

In a second subgroup analysis, PROMS data using the Oxford hip score for those undergoing a DAIR were collected and compared with a concurrent cohort of non-infected patients for quality of outcome measure comparison by one surgeon (author PML).

## Methods

This study reports the results of the DAIR procedures undertaken for all patients having undergone THR, hip hemiarthroplasty or TKR under the care of two surgeons from August 2012 to January 2018. There were no exclusions and no patients lost to follow-up. All data were collected prospectively and analysed retrospectively. All patients had either undergone their index joint replacement under the care of the two surgeons or were referred and admitted under their care from other teams/hospitals. Ideally the timing of the DAIR procedure was agreed to be undertaken within three weeks of onset of symptoms (or delayed wound healing) following their primary surgery. In practice all but one patient underwent their DAIR procedure within this 21-day period (one patient underwent DAIR on day 22 for logistical reasons).

Each DAIR procedure was undertaken by one of the two study surgeons. It entailed opening the existing scar and taking at least five culture samples for culture/sensitivity and histology. Ideally antibiotic treatment was withheld prior to these samples being taken and even in those patients who had commenced antibiotics, treatment was stopped in advance of the DAIR procedure. The operation for each patient was identical and included the removal of modular components, a thorough debridement undertaken followed by irrigation and then replacement of exchangeable modular components. All wounds were closed primarily without drains.

Once the culture samples were taken, IV therapy was commenced (usually IV teicoplanin and gentamicin) or as determined by the multidisciplinary team (MDT) discussion and advised by the consultant microbiologist, who had knowledge of all preoperative circumstances and any antibiotic therapy already given to the patient. Subsequent antibiotic therapy was directed by culture results with initial cultures available within 48 hours and extended cultures at ten days. Most patients were discharged home on IV antibiotics at between 10 and 14 days. IV antibiotics were made possible in the community by trained district nurses. Early and close supervision was undertaken by one of the two consultants to ensure patients remained in remission. IV antibiotics were converted to an oral regime as documented in Table 2. All patients received an extended antibiotic course even when cultures proved negative.

For all patients under the care of one surgeon (PML), Oxford scores were collected prospectively prior to and following their DAIR treatment in order to compare the DAIR group with that same consultant’s non-infected hip and knee arthroplasty cases.

Demographic information including age, gender and BMI was recorded for all patients. Data of infective symptoms and indication for DAIR were also documented along with implant-related information culture and antibiotic regimen. All patients were followed up for a minimum of 67 months on both a clinical and radiological basis. Inflammatory markers were collected, and patients were followed up indefinitely, or until death, for any signs of reinfection.

## Results

This study included 25 patients, 14 of whom were male (Table 1), with a mean age of 72 years (59–83). The average time from onset of symptoms to surgical DAIR was 11 days (range 1–22 days). One patient underwent DAIR 22 days after the onset of symptoms due to logistical delays.

**Table 1:** Primary operation, timing of debridement, antibiotics and implant retention (DAIR) and indication

Case	Age	Gender (M/F)	Primary operation	Duration between index procedure/duration of symptoms and DAIR (days)	Indication for DAIR	Number of months of follow-up	Outcome
1	73	M	TKR	11	Persistent wound ooze/evacuation of haematoma	87	No recurrence of infection
2	67	F	THR	14	Persistent wound ooze	91	No recurrence of infection
3	71	F	THR	14	Persistent wound ooze/evacuation of haematoma	9	No recurrence of infection
4	69	M	THR	16	Persistent wound ooze	95	No recurrence of infection
5	75	M	THR	21	Persistent wound ooze	1 (Died on ITU)	N/A
6	64	M	THR	15	Persistent wound ooze	96	No recurrence of infection
7	76	F	Revision THR	21	Persistent wound ooze	77	No recurrence of infection
8	67	M	THR	13	Evacuation of haematoma	104	One stage revision for aseptic loosening
9	72	M	THR	4	Persistent wound	79	No recurrence of infection
10	62	F	TKR	3	Persistent wound ooze	91	No recurrence of infection
11	70	M	TKR	2	Fever and persistent wound ooze	72	No recurrence of infection
12	79	F	Hemiarthroplasty	16	Persistent wound ooze	83	No recurrence of infection
13	69	M	THR	4	Collection over wound	78	No recurrence of infection
14	83	M	THR	9	Evacuation of haematoma	79	No recurrence of infection
15	73	F	THR	15	Persistent wound ooze and evacuation of haematoma	69	No recurrence of infection
16	83	F	Hemiarthroplasty	9	Wound dehiscence	37	Single stage cemented revision hemiarthroplasty
17	62	F	THR	9	Persistent wound discharge	60	No recurrence of infection
18	68	F	THR	20	Persistent wound ooze	67	No recurrence of infection
19	75	F	TKR	11	Persistent wound ooze	40	Revision to knee arthrodesis
20	79	M	THR	9	Palpable collection	66	No recurrence of infection
21	68	M	THR	9	Palpable collection	66	No recurrence of infection
22	59	M	Hemiarthroplasty	1	Evacuation of haematoma	50	No recurrence of infection
23	54	M	THR	5	Evacuation of haematoma	50	No recurrence of infection
24	87	M	THR	2	Leaking wound	32	No recurrence of infection
25	70	M	THR	22	Leaking wound	23	No recurrence of infection

TKR = total knee replacement ; THR = total hip replacement

The index procedure for each patient and indication for DAIR are summarised in Table 1 and involved four TKRs, 17 THRs, one revision THR and three hip hemiarthroplasties. Table 2 documents the microorganisms cultured and duration of antibiotic treatment. There were no negative cultures in the primary THR group with 8/17 growing multiple organisms and the remaining 9 growing a single organism. In the TKR group, one patient remained culture negative, two grew a single organism and one multiple organisms.

**Table 2:** Cultured microorganisms and antibiotic therapy

Case	Cultured micro-organism	Antibiotics (IV and oral)	Total duration of antibiotic therapy (months)
1	Nil	IV: teicoplanin, ciprofloxacin, clindamycin Oral: metronidazole	3
2	*Staphylococcus epidermidis*	IV: teicoplanin and rifampicin	3
3	*Staphylococcus aureus*, *Enterococcus faecium*	IV: teicoplanin, linezolid Oral: Pristinomycin	6
4	*E. coli*, *Enterococcus faecalis*	IV: teicolplanin, tazocin Oral: ciprofloxacin	3
5	*Staphylococcus epidermidis*, *Proteus mirabilis*	Died on admission in ITU	Died on admission in ITU
6	*Staphylococcus aureus Staphylococcus epidermidis*	IV: teicolplanin Oral: rifampicin	3
7	Nil	IV: flucloxacillin, benzylpenicillin, teicoplanin, rifampicin Oral: rifampicin, doxycycline	6
8	Coagulase negative *Staphylococcus, anaerobes*	IV: ertapenem, vancomycin, augmentin and tazocin Oral: ciprofloxacillin and rifampicin	6
9	*Staphylococcus epiderimidis*	IV: teicoplanin, vancomycin Oral: rifampicin	6
10	*Staphylococcus aureus*	IV: flucloxacillin Oral: ciprofloxacin, rifampicin	3
11	*Streptococcus agalactiae*	IV: teicoplanin,ceftriaxone, benzylpenicillin Oral: rifampicin	4
12	*E. coli*	IV: teicoplanin Oral: ciprofloxacin, flucloxacillin	3
13	*E. coli*	IV: amoxicillin Oral: ciprofloxacin	3
14	*Staphylococcus aureus*	IV: flucloxacillin Oral: rifampicin, ciprofloxacin	3
15	*Enterococcus faecalis*, *Staphylococcus epidermidis*, *Citrobacter kaseri*	IV: daptomycin, ciprofloxacin Oral: ciprofloxacin, linezolid	4
16	*Staphylococcus epidermidis*	IV: vancomycin Oral: rifampicin, linezolid, doxycycline	3
17	*Staphylococcus aureus*, Pseudomonas	IV: flucloxacillin, rifampicin Oral: rifampicin, ciprofloxacin	4
18	*E. coli*, *Staphylococcus hummis*, *Enterococcus faecalis*, *Proteus*	IV: teicoplanin Oral: ciprofloxacin	2
19	*Proteus*, *Staphylococcus epidermidis*	IV: teicoplanin, vancomycin, tazocin Oral: ciprofloxacin	4
20	*E. coli*	IV: ciprofloxacin Oral: ciprofloxacin	3
21	*E. coli* and *Enterococcus*	IV: tazocin Oral: ciprofloxacin	3
22	*Staphylococcus epidermidis*	IV: teicoplanin Oral: doxycycline, rifampicin	3
23	Mixed coagulase negative *Staphylococci*	Amoxicillin	3
24	*Staphylococcus aureus*	IV: teicoplanin, rifampicin Oral: ciprofloxacin, rifampicin	3
25	*Corynebacterium jeikeium*	Linezolid	3

Thirteen out of twenty-five (52%) of patients received IV teicoplanin for a mean duration of 3.8 months and 4/25 (16%) received IV flucloxacillin as determined by the cultures. The remaining patients were treated with a regime determined by the involved consultant microbiologist.

Nineteen of the twenty-five patients (76%) made an uneventful recovery without evidence of recurrent infection either in the immediate recovery or later outpatient follow-up. Serial CRP and x-rays remained satisfactory, and all patients remain on annual follow-up. There were no patients lost to follow-up. Average follow-up to date was 67 months (range 9–104 months).

Four out of the 25 patients have required further surgical intervention:

**Patient 1** required intervention for aseptic loosening of their THR 6.5 years after DAIR procedure. He underwent a single-stage revision with all intraoperative culture samples negative and remains well.
**Patient 2** underwent DAIR following TKR at 12 days post index surgery for persistent wound discharge. Cultures grew *Proteus* and *Staphylococcus epidermidis* and despite treatment, this patient continued to have a persistent wound discharge and underwent a knee arthrodesis 2 months following the DAIR.
**Patient 3** underwent a DAIR at 21 days following hip hemiarthroplasty with positive growth of *S. epidermidis* on cultures and persistent wound ooze. A single-stage cemented revision hemiarthroplasty was performed due to persistent ooze, and the patient has remained asymptomatic and free of infection at last follow-up.
**Patient 4** underwent DAIR 2 years following primary total hip replacement. They presented with a further acute onset of pain, wound discharge and effusion on MRI. They underwent a two-stage revision and is the only patient requiring a two-stage intervention in this series. Cultures grew *Escherichia coli* and the patient remains asymptomatic 48 months following the second stage re-implantation.

An additional interesting finding is the PROMS analysis undertaken for six primary THR patients who underwent DAIR in the immediate postoperative period. The results for these patients were compared with the THR database of 792 patients for the same surgeon. At six weeks postoperative, mean OHS was 31.9 for the DAIR group compared with 34.0 for the controls. By one year, the OHS results remain comparable at 36.0 and 39.0 for DAIR and the controls, respectively. One could argue this is a small subgroup analysis, but for two reasons these OHS findings are important. Firstly, these six patients represent 24% (6 of 25) of all our DAIR PJIs treated during the study period and without any data or patient loss. Secondly, although the numbers are small, PJI invariably represents only a small number of arthroplasties. To obtain larger numbers with PJI, it would require at least a multicentre study with the inevitable introduction of variation in hospitals, surgeons and techniques. For these reasons we include these findings for discussion.

Longer-term patient follow-up has documented eight deaths, seven of which were unrelated to their arthroplasty treatment. Seven patients died of medical causes that included cerebral vascular accident, myocardial infarction, etc., and the average survival following DAIR was 33 months (1–77 months). The final patient presented with a persistent wound ooze following primary THR. This was initially managed conservatively, but the patient fell on the ward while rehabilitating and sustained an undisplaced Vancouver B periprosthetic fracture. Six days later, he developed sepsis and the wound ooze did not settle. He returned to theatre and had a DAIR (cultures grew *Proteus mirabilis* and *S. epidermidis*). During the postoperative period he was transferred to intensive care, but failed to respond to treatment, continued to deteriorate and died (cause of death documented as pericarditis).

**Figure 1:**
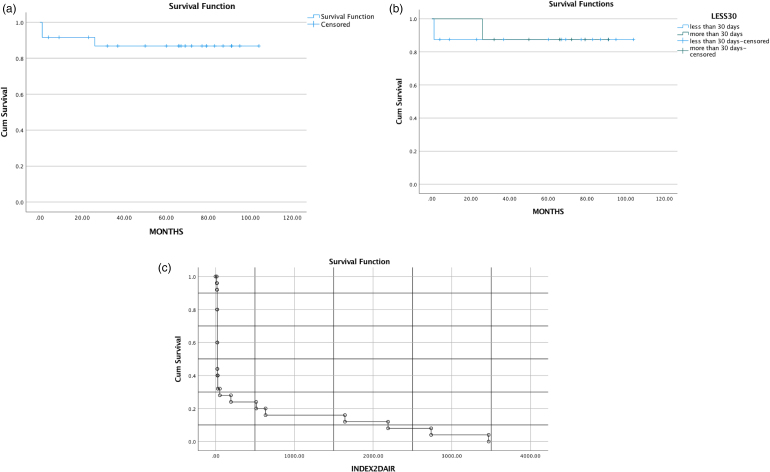
Survival analysis and Kaplan–Meier curves. (a) Survival analysis with failure of debridement, antibiotics and implant retention (DAIR) as event. (b) Comparative analysis with duration between index surgery and DAIR (<30 days vs >30 days); log rank test shows *p*-value = 0.915. (c) Survival analysis of DAIR and duration between index procedure and DAIR procedure. Survival curve with event as DAIR (y-axis) and duration between index procedure and DAIR in x-axis. Inference in our study was that the majority of DAIR (68%) happened in less than 30 days from the index procedure. IBM SPSS Statistics Version 27.0.1.0.

Finally, a survival analysis was carried out using Kaplan–Meier curves for the following domains: survival analysis with failure of DAIR as event; comparative analysis with duration between index surgery and DAIR (<30 days vs >30 days) and survival of the DAIR and duration between index procedure and DAIR (Figure 1).

## Discussion

This study was undertaken to document the incidence and outcome of DAIR procedures from a busy arthroplasty unit but without a specialist interest in joint infection. To reduce any variation due to surgeon preference or practice, the results were obtained from only two surgeons from the same trust and following an agreed strict protocol including timing of intervention, antibiotic use/duration and patient follow-up. Antibiotic use, its type and duration of treatment was also carefully supervised by one consultant microbiologist.

Only since 2018 has the DAIR procedure become a notifiable operation/procedure in the National Joint registry and as described in the introduction to this paper, variation exists as to the procedure itself, its timing and its effectiveness.^11,12^ Firstly, regarding the operation, data do exist to favour modular implant exchange. Choi *et al* retrospectively reviewed 75 patients who underwent a total knee replacement with subsequent infection and compared patients who were treated with incision and drainage (+/− polyethylene (PE) exchange) or a two-stage revision procedure.^5^ Choi *et al* found that infection control was lower in the retention group compared with the two-stage revision (31% vs 59%, respectively). The two main factors for failure in the retention group were attributed to *S. aureus* infection and lack of PE exchange, and that the lack of PE exchange appeared to account for a poor outcome irrespective of the causative organism.

A failure to exchange modular components resulting in an increased failure rate has also been reported in other studies. Grammatopoulos *et al* analysed all hip DAIR procedures performed in their unit over an 18-year period. They too found that failure to exchange modular components resulted in a three-fold increase in the failure rate.^13^ Therefore exchanging modular components when performing DAIR appears to be a key step in minimising reinfection or failure to eradicate infection. This may be in part due to having better access to the posterior aspect of the joint in TKR and therefore a more thorough debridement.^12^

Secondly, the timing of DAIR has been variable in many studies. In ours, postoperative arthroplasty patients were eligible for DAIR when presenting with relevant symptoms up to three weeks following index surgery and in those with suspected late haematological infection, with the aim of also performing their DAIR within a similar three-week period. Only one patient in our cohort was operated on the 22nd day after the onset of symptoms. All other patients presenting to our unit after this time were not considered suitable for DAIR, and where necessary a two-stage revision was undertaken.

Another publication by Zimmerli *et al* proposed in their original paper (consistent with our study policy for early surgery)^14^ that DAIR should only be performed within three weeks after a haematogenous infection and within four weeks for postoperative infections. The three-week cut-off has been adopted by several units and shown to improve outcomes compared with longer durations.^15^

Laffer *et al* compared DAIR with two-stage revisions confined to TKR and found that the success rate was similar for the two treatments for patients presenting with early and late haematogenous infections with a short duration of symptoms (<3 weeks).^15^ Further support for early intervention has been reported by Qu *et al* who analysed 1,266 prosthetic knee infections and found that the success rate halved when surgery was delayed more than three weeks from onset of symptoms.^16^ Similar findings have been replicated in other units^17–19^ and most recently in a study by Veerman *et al*, which demonstrated an increased failure rate when DAIR was performed more than 30 days after the index procedure and when DAIR was repeated within 90 days within a two-year period of follow-up.^20^

So why should DAIR be successful? The most widely theoretically accepted explanation for the success of DAIR in early PJI is that bacteria are still in the ‘planktonic’ phase and are therefore more readily managed with the host immune response and antibiotics.^21^ Once infections present late, a biofilm is said to form, which is a stationary growth phase that is more resistant to antibiotic therapy and presumably this necessitates more aggressive surgical treatment.^6^

Unfortunately defining the exact onset of infective symptoms can be challenging and subjective, which can often result in treatment delay. In our study, all suspected PJIs were discussed in an MDT meeting involving both surgeons and a specialist microbiologist, and patients were closely monitored clinically and biochemically. This offered a strict standardisation of care and may also explain the variations in inclusion criteria in other studies, particularly when patients are reviewed by multiple different teams^22^ with varying opinions, protocols and experience.

In our study all patients received a minimum of two months of antibiotics using a combination of intravenous and oral regimes. Only one PJI suffered persistent infection and required a two-stage revision. The duration of antibiotics is again a controversial topic, and at present there appears to be little consensus for their duration, be it oral or intravenous. Chaussade *et al*^23^ retrospectively reviewed 87 patients with PJI and found that there were no significant differences in remission rates between patients receiving 6 or 12 weeks of antibiotics. These findings were also documented by others; for example, Bernard *et al* found no statistically significant difference in remission rate related to antibiotic duration.^24^ However, Byren *et al* in another retrospectively analysed study of DAIR procedures over a five-year period found that the majority of treatment failures occurred in the first four months after stopping the antibiotics.^16^ Therefore, although there seems to be little consensus on antibiotic duration, withholding antibiotics after DAIR appears both illogical and more likely to result in failure. Yange *et al*^25^ also highlighted the importance of culture-directed antibiotic therapy following a randomised controlled trial on 185 patients who either received a 3-month course of microorganism-directed oral antibiotics (after re-implantation of a second stage hip/knee arthroplasty) versus a control group who did not receive antibiotics. The aim of their study was to analyse the role of microorganism-directed oral antibiotics after discharge from hospital and the role of extended antibiotics in preventing further infection for a two-year period. They found a highly significant 9 out of 72 (12.5%) patients in the antibiotic group had a reinfection compared with 20 out of 70 patients in the control group (*p* = 0.012).

One of our patients following hip hemiarthroplasty required a single-stage revision to a cemented hemiarthroplasty. The success of DAIR after hemiarthroplasty has been shown to be less successful when compared with THR.^26–28^ These authors postulate that this may be due to patient-related factors such as the presence of chest infections, pressure sores, urinary tract infections or other concomitant infections.^26^ In another study, Craxford *et al* also suggested that this recurrent infection may be related to the type of organisms cultured. Our patient grew *S. epidermidis*, but in their series, patients with a gram-positive organism (single organism) had only a 27.8% success rate (prevention of recurrence of infection) after DAIR.^26^

One interesting and important factor that may pose a therapeutic challenge is the culture-negative PJI.^29^ In our study, one revision THR and one TKR patient had a culture-negative PJI. We felt in order to minimise the number of culture-negative PJIs, where possible, antibiotics should be withheld pre- and intraoperatively until samples had been obtained, and ensuring that extended cultures were performed. Both these culture-negative patients received empirically a broad spectrum of antibiotics after the DAIR procedure, i.e. treated as infected. The TKR patient was treated with three months of antibiotics (teicoplanin, ciprofloxacillin, clindamycin, metronidazole) and the revision THR patient with six months of antibiotics (flucloxacillin, teicoplanin, rifampicin, doxycycline), and both patients remain in remission (87 months and 77 months follow-up, respectively).

Finally, we analysed the midterm clinical outcome of DAIR using PROMS for six THR patients. These outcomes have been documented by Grammatopoulos *et al*.^30^ In their analysis of the outcomes of 82 patients undergoing DAIR performed at their institution, patients were compared with two control groups (a primary THR and two-stage revision group). They reported a 98%, five-year survival rate of successful DAIR procedures (similar to the primary THR group), and first-time DAIR procedures had no statistically worse OHS scores compared with the control THR group. Our findings are the same. Their mean follow-up was three years longer than our study, but our data support the success of this intervention for the medium term.

Our study has some limitations. Firstly, thankfully, despite a large volume of arthroplasty procedures undertaken, PJI was rare and accounted for only a small sample size. However, this sample size is consistent with the workload of many busy orthopaedic units. One major advantage of our study was all DAIR procedures were performed by only one of two surgeons, both adhering to a standardised protocol. Lastly, the average follow-up was 5 years (67 months) (range 9–104 months) and data collection continues for long-term results.

## Conclusion

This study demonstrates that 21 of 25 patients were successfully treated with DAIR at a non-PJI specialist centre with only one case of reinfection, over a five-year follow-up period. A strictly defined DAIR protocol and procedure (including modular implant exchange) appears to be a successful treatment strategy for the management of early PJI and avoids the need for a more major surgical intervention, costly and socially demanding hospital transfer, and/or lifelong suppressive therapy. An MDT approach is advocated, involving a small number of interested surgeons and microbiologists focusing on prompt surgery and sensitivity-directed extended course antimicrobial therapy.
